# Supplementary material for: Regionalization of gene expression and cell types in the silk gland of the pantry moth Plodia interpunctella
Source: bioRxiv. 2025 Jul 16:2025.07.11.664249. Preprint. [Version 1] doi: 10.1101/2025.07.11.664249 (PMC12338581; doi:10.1101/2025.07.11.664249)
Supplement: Supplement 5 [file NIHPP2025.07.11.664249v1-supplement-5.pdf]

bioRxiv preprint doi: https://doi.org/10.1101/2025.07.11.664249; this version posted July 16, 2025. The copyright holder for this preprint (which was not certified by peer review) is the author/funder, who has granted bioRxiv a license to display the preprint in perpetuity. It is made available under aCC-BY 4.0 International license.

Supplementary Information

Figure S1: DAPI and phalloidin staining of silk gland compartments
Figure S2: Homology and microsynteny of *Ser1* sericin genes in Lepidoptera
Figure S3: Homology and microsynteny of *SerP150* and *Mucin12* sericin factor genes in Lepidoptera
Figure S4: Homology of *MG4* sericin factor genes in Pyraloidea
Figure S5: Homology of *Ser3* sericin genes in Pyraloidea
Figure S6: Homology and microsynteny of seroin genes in Lepidoptera
Table S1: HCR Oligonucleotides Sequences
Table S2 : DESeq2 Results Table
Table S3 : Table containing the DESeq2 normalized counts for all annotated genes in the *ilPloInte3.2* genome
Table S4 : Table containing data plotted in the Heatmap, related to Figure 3
Table S5 : Table containing the TPM normalized counts for all annotated genes in the *ilPloInte3.2* genome
Table S6 : Table containing data plotted in the Scatter Plot, related to Figure 3
Table S7 : Accession numbers for sericin factor genes used in synteny analyses and alignments
Table S8: Amino acid and signal peptide sequences for top MSG and PSG enriched genes
Table S9: Manually Curated *ilPloInte3.2* Gene List
File S1: Manually Curated *ilPloInte3.2* Genome Annotation GTF
Video S1: 3D rendering of silk glands in a fifth instar moth larva visualized by micro-CT.. Related to Figure 1
Video S2: XY-plane micro-CT slices through a fifth instar moth larva.
Animated sequence of consecutive 2D micro-CT sections scrolling through the larva from anterior to posterior. Slices highlight internal anatomical structures including the changes in diameter, looping, and crossing of the silk glands along the larva's body axis. Related to Figure 1.
Video S3: 3D projection of DAPI, Phalloidin, and WGA stainings, from confocal microscopy stacks of the ASG, MSG, and PSG , related to Figure 1

bioRxiv preprint doi: https://doi.org/10.1101/2025.07.11.664249; this version posted July 16, 2025. The copyright holder for this preprint (which was not certified by peer review) is the author/funder, who has granted bioRxiv a license to display the preprint in perpetuity. It is made available under aCC-BY 4.0 International license.

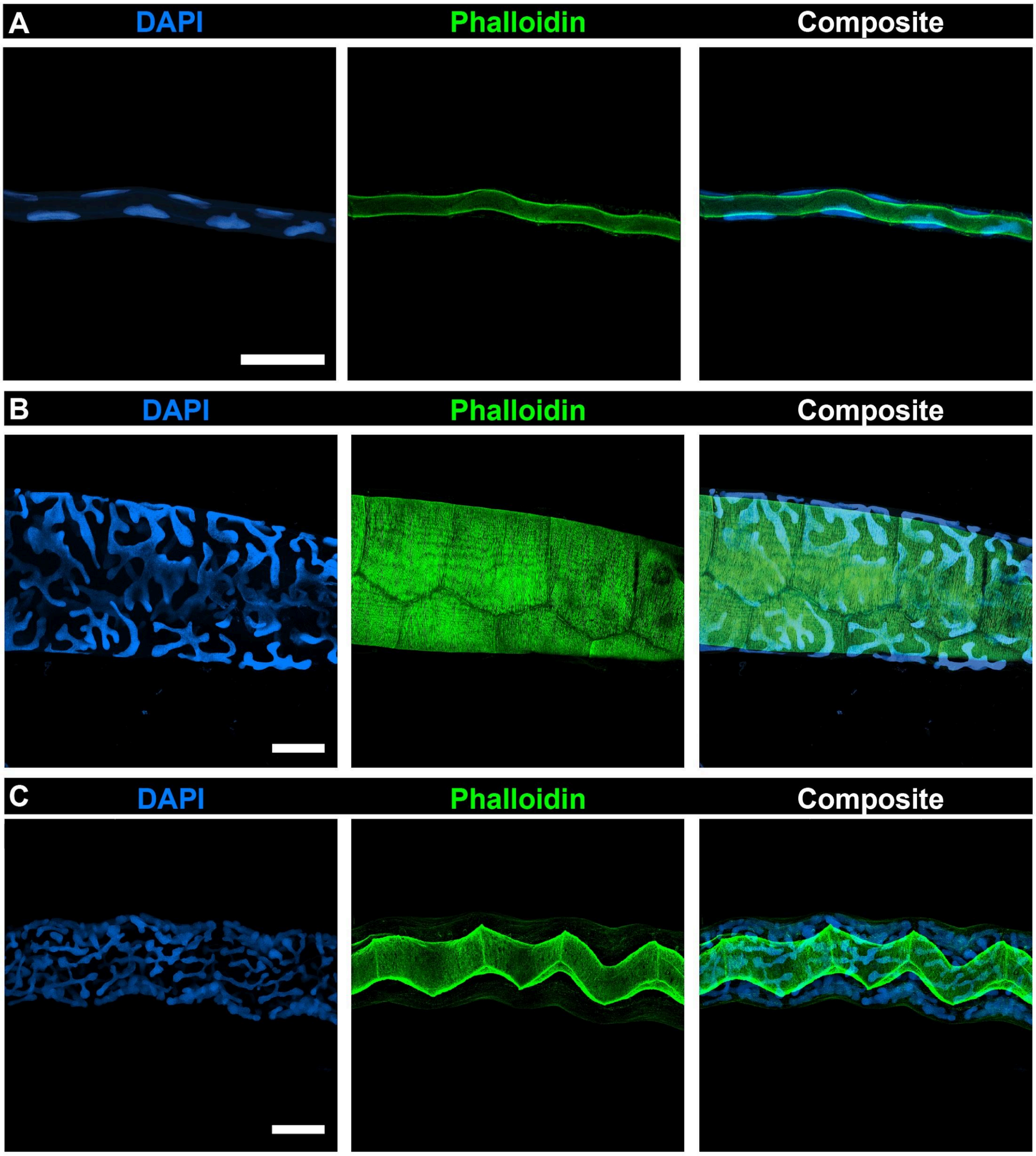

Figure S1. DAPI and Phalloidin staining reveal striking differences in cellular morphology and arrangement between compartments. A-C. DAPI and phalloidin staining of an ASG (A), MSG (B), and PSG (C). See Video S3 for a video exploring the 3D projections of these images. The images from panel A-B are taken of the same gland while the images in panel C are taken of a different gland from an individual of the same developmental stage (wandering fifth instar). Scale bars: **A-C** = 100 µm

bioRxiv preprint doi: https://doi.org/10.1101/2025.07.11.664249; this version posted July 16, 2025. The copyright holder for this preprint (which was not certified by peer review) is the author/funder, who has granted bioRxiv a license to display the preprint in perpetuity. It is made available under aCC-BY 4.0 International license.

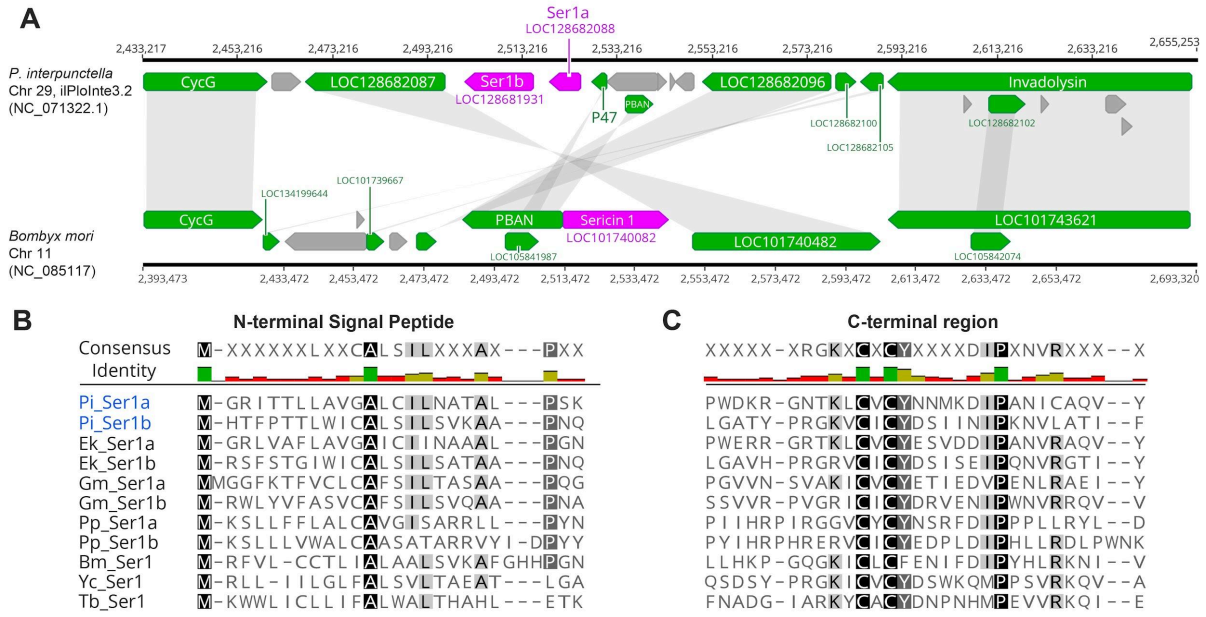

Figure S2. Homology of *Ser1* sericin gene copies across Lepidoptera. A. Synteny analysis reveals *Ser1a* and *Ser1b* genes of pyralid genomes (here on top, *P. interpunctella*) are found as tandem duplicates, within an inverted syntenic block containing the *B. mori Ser1* homolog (magenta). Grey fields indicate sequence matches using reciprocal TBLASTN between the predicted protein of a first species and the NCBI RefSeq_RNA dataset of the second species. **B-C.** Protein alignments of Ser1 syntenologs from genome annotations of lepidopteran species, largely based on previous analyses [10,41,105,106], with a focus on the N-terminal signal peptide (B) and the C-terminal region (C). The C-terminal CxCx motif is unique to this sericin orthology group. Gene and protein identifiers are listed in **Table S7**. Ek : *Ephestia kuehniella* (Pyralidae, Phycitinae), *Plodia interpunctella* (Pyralidae, Phycitinae); Gm : *Galleria mellonella* (Pyralidae, Galleriinae); Pp : *Pseudoips prasinana* (Nolidae; Chloephorinae); Bm : *Bombyx mori* (Bombycidae, Bombycinae); Yc : *Yponomeuta cagnagella* (Yponomeutidae; Yponomeutinae) ; Tb : *Tineola bisselliella* (Tineidae; Tineinae).

bioRxiv preprint doi: https://doi.org/10.1101/2025.07.11.664249; this version posted July 16, 2025. The copyright holder for this preprint (which was not certified by peer review) is the author/funder, who has granted bioRxiv a license to display the preprint in perpetuity. It is made available under aCC-BY 4.0 International license.

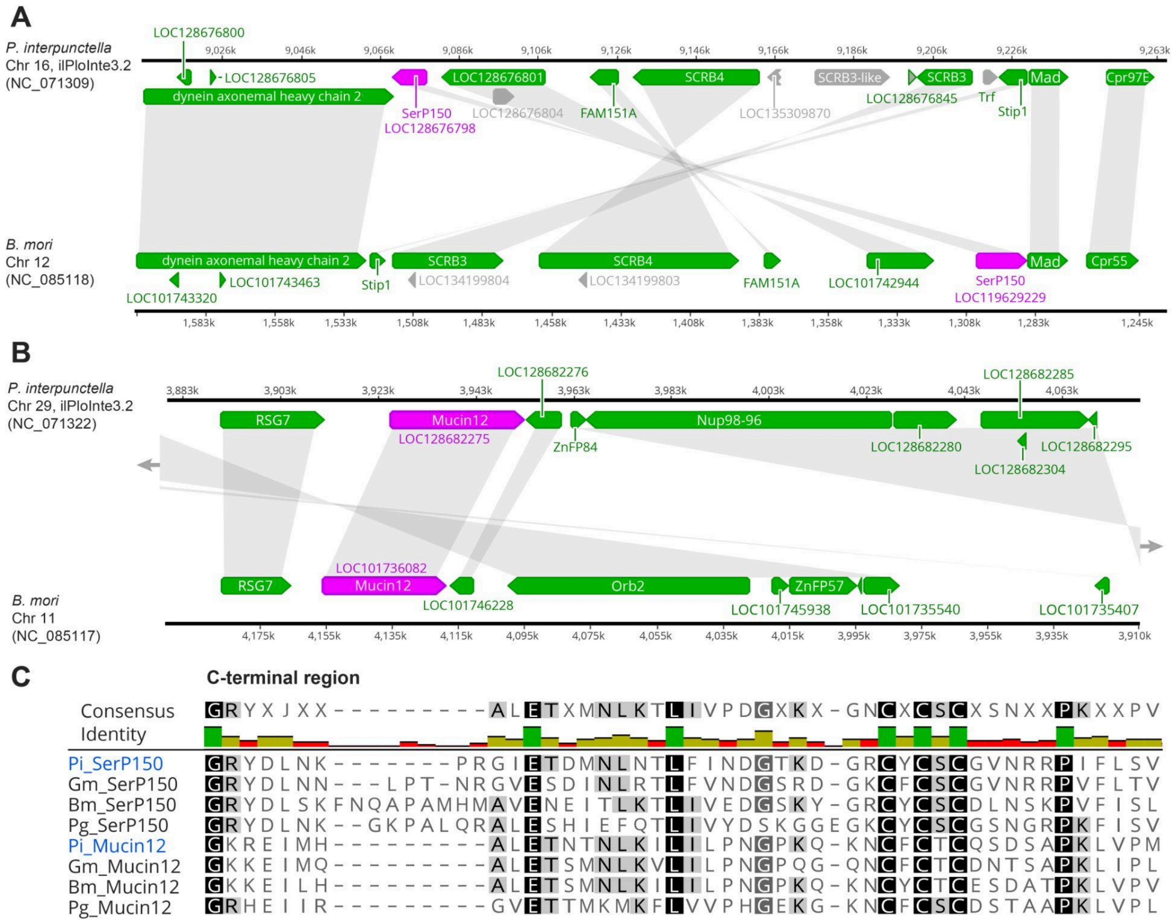

Figure S3. Homology and microsynteny of *SerP150* and *Mucin12* sericin gene orthologues in Lepidoptera. A-B. Microsyntenic relationships of the *SerP150* (A), based on a previous study[69], and *Mucin12* (B) gene regions between *P. interpunctella* (top) and *B. mori*. Grey fields indicate sequence matches using reciprocal TBLASTN between the predicted protein of a first species and the NCBI RefSeq_RNA dataset of the second species. **C.** Protein alignments of SerP150 and Mucin12 syntenologs from various lepidopteran insects, characterized by a conserved CxCxC motif in their C-terminal domains. This alignment replicates the findings of a previous study[69]. Gene and protein identifiers are listed in **Table S7**. Pi : *Plodia interpunctella* (Pyralidae); Gm : *Galleria mellonella* (Pyralidae); Bm : *Bombyx mori* (Bombycidae); Pg : *Pectinophora gossypiella* (Gelechiidae).

bioRxiv preprint doi: https://doi.org/10.1101/2025.07.11.664249; this version posted July 16, 2025. The copyright holder for this preprint (which was not certified by peer review) is the author/funder, who has granted bioRxiv a license to display the preprint in perpetuity. It is made available under aCC-BY 4.0 International license.

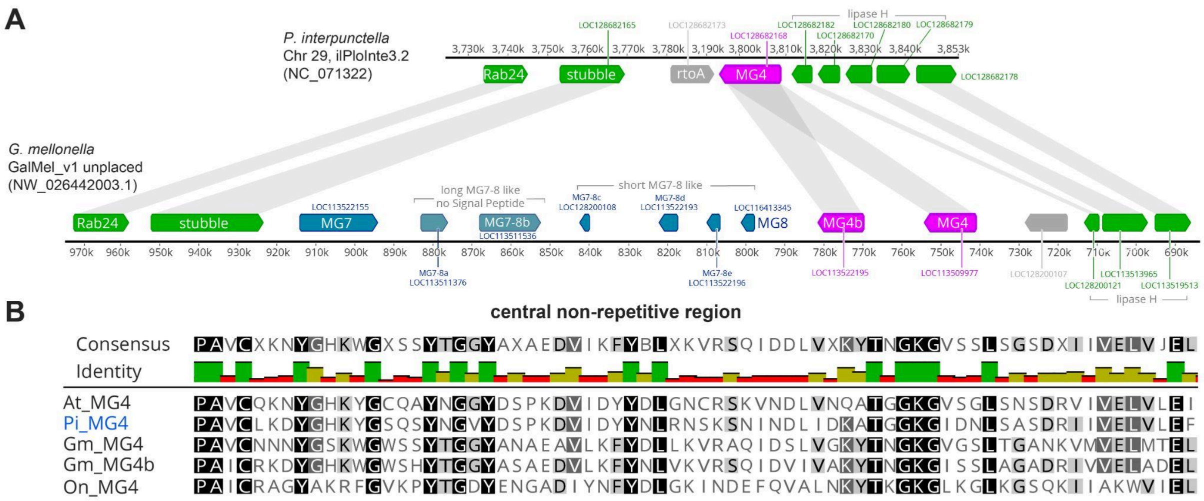

Figure S4. Homology of MG4 sericin factors in Pyraloidea. A. Synteny comparison between gene regions encompassing a single copy of the *MG4* sericin factor in *P. interpunctella* (top), and a cluster of related MSG-enriched genes specific to the *G. mellonella* genome, as previously described[10,12,107,108]. Grey fields indicate sequence matches using reciprocal TBLASTN between the predicted protein of a first species and the NCBI RefSeq_RNA dataset of the second species. *G. mellonella MG7-MG8* genes are likely paralog copies of *MG4*, consistent with an expansion of sericin genes in this species. **B.** Protein alignments of MG4 homologs from genome annotations of pyralid and crambid species (Pyraloidae), with a focus on the central domain, N-terminal to a serine-rich repeat region). Gene and protein identifiers are listed in **Table S7**. At : *Amyelois transitella* (Pyralidae, Phycitinae); Pi : *Plodia interpunctella* (Pyralidae, Phycitinae); Gm : *Galleria mellonella* (Pyralidae, Galleriinae); On : *Ostrinia nubilalis* (Crambidae, Pyraustinae).

bioRxiv preprint doi: https://doi.org/10.1101/2025.07.11.664249; this version posted July 16, 2025. The copyright holder for this preprint (which was not certified by peer review) is the author/funder, who has granted bioRxiv a license to display the preprint in perpetuity. It is made available under aCC-BY 4.0 International license.

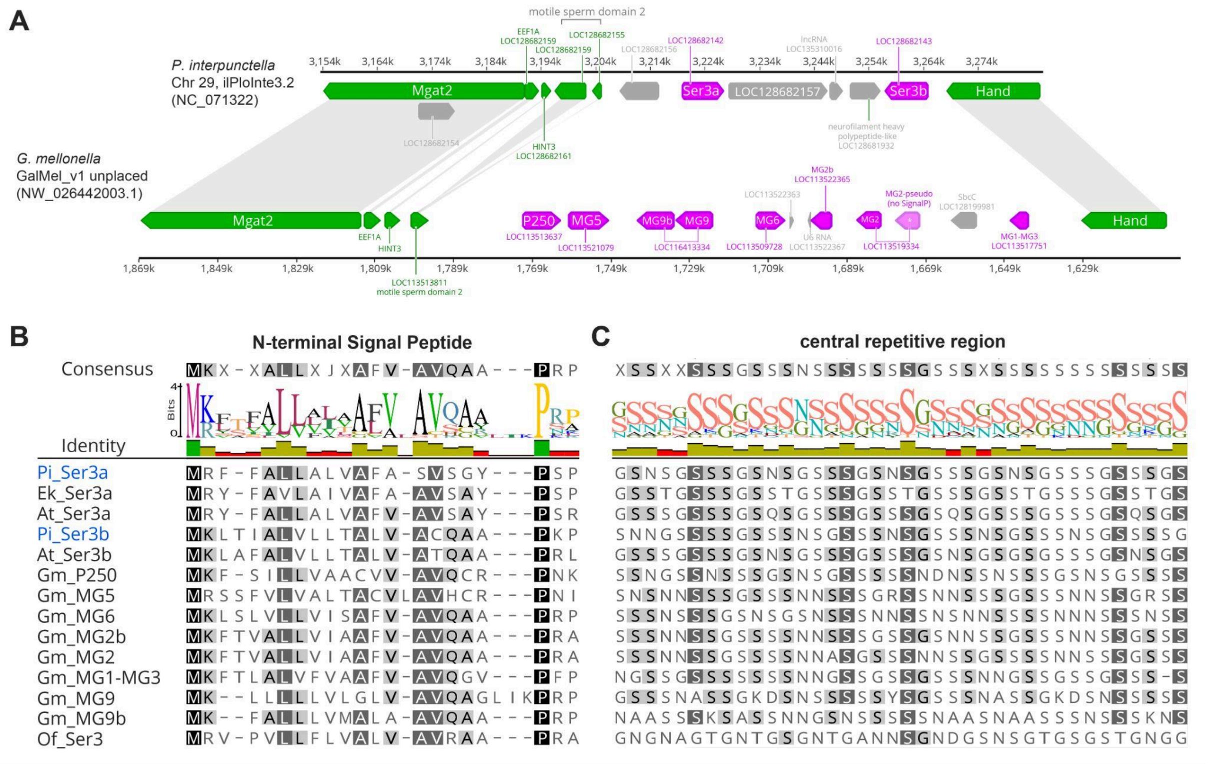

Figure S5. Homology of *Ser3* sericin factors in Pyraloidea. A. Synteny comparison between gene regions encompassing two copies of the *Sericin3* gene in *P. interpunctella* (top), hereby dubbed *Ser3a* and *Ser3b*, and a cluster of related MSG-enriched genes specific to the *G. mellonella* genome[10,12,107,108]. Grey fields indicate sequence matches using reciprocal TBLASTN between the predicted protein of a first species and the NCBI RefSeq_RNA dataset of the second species, but are only shown for flanking syntenic genes (green). Ser3 homologs (magenta) all show a high degree of similarity dominated by serine-rich repeats. **B-C.** Protein alignments of Ser3 orthologs from genome annotations of pyralid and crambid species (Pyraloidae), including copies of Ser3a previously dubbed as Ser3 in *E. kuehniella* and *A. transitella*[10], with a focus on the N-terminal signal peptide (B) and a central repetitive region (C). Gene and protein identifiers are listed in **Table S7**. At : *Amyelois transitella* (Pyralidae, Phycitinae); Ek : *Ephestia kuehniella* (Pyralidae, Phycitinae); Pi : *Plodia interpunctella* (Pyralidae, Phycitinae); Gm : *Galleria mellonella* (Pyralidae, Galleriinae); Of : *Ostrinia furnacalis* (Crambidae, Pyraustinae).

bioRxiv preprint doi: https://doi.org/10.1101/2025.07.11.664249; this version posted July 16, 2025. The copyright holder for this preprint (which was not certified by peer review) is the author/funder, who has granted bioRxiv a license to display the preprint in perpetuity. It is made available under aCC-BY 4.0 International license.

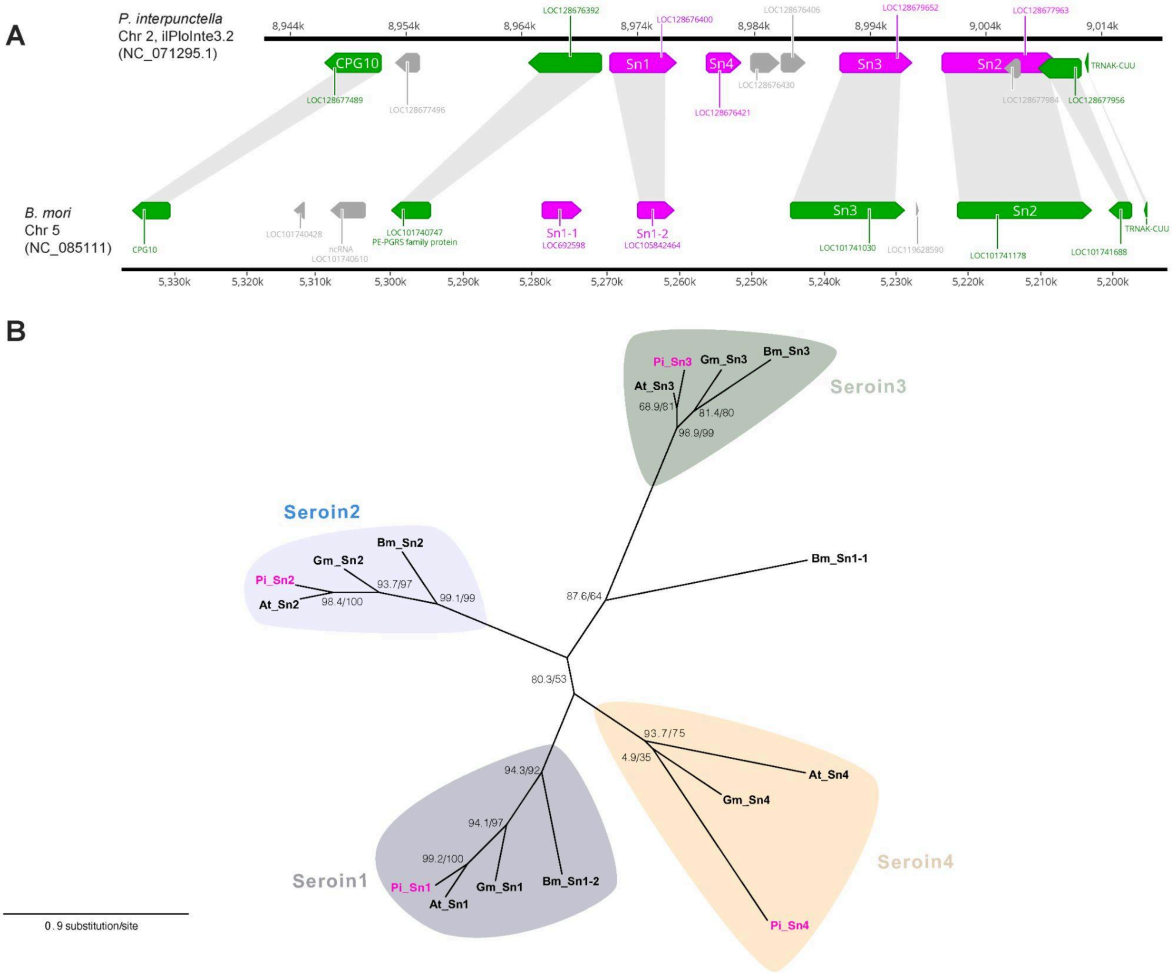

Figure S6. Homology and microsynteny of seroin genes in Lepidoptera. A. Synteny comparison between the clusters encompassing four seroin genes in *P. interpunctella* (top) and *B. mori* (bottom). Grey fields indicate sequence matches using reciprocal TBLASTN between the predicted protein of a first species and the NCBI RefSeq_RNA dataset of the second species. **B.** Maximum likelihood phylogenetic reconstruction of pyralid and *B. mori* seroin proteins, highlighting four orthology groups. Branch support is indicated by SH-aLRT % values / ultrafast bootstrap % values. Gene and protein identifiers are listed in **Table S7**. At : *Amyelois transitella* (Pyralidae, Phycitinae); Bm : *Bombyx mori* (Bombycidae, Bombycinae) ; Gm : *Galleria mellonella* (Pyralidae, Galleriinae) ; Pi : *Plodia interpunctella* (Pyralidae, Phycitinae).
